# Constructing Binder‐ and Carbon Additive‐Free Organosulfur Cathodes Based on Conducting Thiol‐Polymers through Electropolymerization for Lithium‐Sulfur Batteries

**DOI:** 10.1002/cssc.202200434

**Published:** 2022-05-30

**Authors:** Jiaoyi Ning, Hongtao Yu, Shilin Mei, Yannik Schütze, Sebastian Risse, Nikolay Kardjilov, André Hilger, Ingo Manke, Annika Bande, Victor G. Ruiz, Joachim Dzubiella, Hong Meng, Yan Lu

**Affiliations:** ^1^ Department for Electrochemical Energy Storage Helmholtz-Zentrum Berlin für Materialien und Energie GmbH Hahn-Meitner Platz 1 14109 Berlin Germany; ^2^ School of Advanced Materials Peking University Shenzhen Graduate School Peking University Lishui road 2199, Nanshan district Shenzhen 518055 P. R. China; ^3^ Guangdong Province Key Laboratory of Durability for Marine Civil Engineering School of Civil Engineering Shenzhen University Shenzhen 518060 P. R. China; ^4^ Research Group Simulation of Energy Materials Helmholtz-Zentrum Berlin für Materialien und Energie GmbH Hahn-Meitner Platz 1 14109 Berlin Germany; ^5^ Institute of Chemistry and Biochemistry Freie Universität Arnimallee 22 14195 Berlin Germany; ^6^ Institute for Applied Materials Helmholtz-Zentrum Berlin für Materialien und Energie GmbH Hahn-Meitner Platz 1 14109 Berlin Germany; ^7^ Theory of Electron Dynamics and Spectroscopy Helmholtz-Zentrum Berlin für Materialien und Energie GmbH Hahn-Meitner Platz 1 14109 Berlin Germany; ^8^ Physikalisches Institut Albert-Ludwigs-Universität Freiburg Hermann-Herder-Straße 3 79104 Freiburg Germany; ^9^ Institute of Chemistry University of Potsdam 14467 Potsdam Germany

**Keywords:** electrochemistry, energy storage, lithium-sulfur batteries, operando studies, organosulfur

## Abstract

Herein, the concept of constructing binder‐ and carbon additive‐free organosulfur cathode was proved based on thiol‐containing conducting polymer poly(4‐(thiophene‐3‐yl) benzenethiol) (PTBT). The PTBT featured the polythiophene‐structure main chain as a highly conducting framework and the benzenethiol side chain to copolymerize with sulfur and form a crosslinked organosulfur polymer (namely S/PTBT). Meanwhile, it could be in‐situ deposited on the current collector by electro‐polymerization, making it a binder‐free and free‐standing cathode for Li‐S batteries. The S/PTBT cathode exhibited a reversible capacity of around 870 mAh g^−1^ at 0.1 C and improved cycling performance compared to the physically mixed cathode (namely S&PTBT). This multifunction cathode eliminated the influence of the additives (carbon/binder), making it suitable to be applied as a model electrode for operando analysis. Operando X‐ray imaging revealed the remarkable effect in the suppression of polysulfides shuttle via introducing covalent bonds, paving the way for the study of the intrinsic mechanisms in Li‐S batteries.

## Graphical abstract

**Free and organic**: A binder‐ and carbon additive‐free organosulfur cathode is constructed through electropolymerization and vulcanization, based on thiol‐containing conducting polymer poly(4‐(thiophene‐3‐yl) benzenethiol) (PTBT). The chemical confinement of polysulfide is achieved with improved cycling performance. This multifunctional cathode can be used as a model electrode for operando analysis.

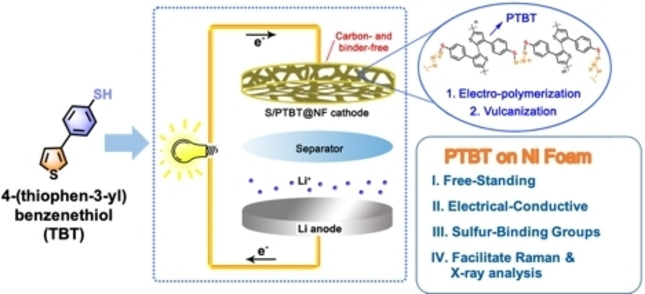

## Introduction

Lithium‐sulfur (Li‐S) batteries, using sulfur as an active cathode material, have attracted considerable attention owing to the superiority of electrode materials, especially the high theoretical capacity (≈1675 mAh g^−1^) of sulfur, which is simultaneously Earth‐abundant, cheap, and environmentally benign.^[1]^ However, this superior battery type also suffers from various drawbacks hindering its commercialization, such as the “shuttle effect” of the soluble lithium polysulfides during the charging‐discharging cycle process, the insulating nature of sulfur and lithium sulfide, and the large volume expansion of sulfur (≈80 %) on its full lithiation.^[2]^ To alleviate these issues for improving the performance of Li‐S batteries, in particular, the exploitation of high‐performance cathode materials is highly desired. So far, plenty of inorganic framework material, such as carbon material (e. g., mesoporous carbon, hollow carbon spheres, carbon nanotubes, and graphene), as well as metal oxides/sulfides/nitrides and metal‐organic frameworks have been designed and fabricated through chemical synthesis methods.^[3]^ They should prevent the loss of the soluble polysulfides through physical/chemical confinement as well as increase the electrical conductivity of cathodes. On the other hand, the chemical confinement, through fixing polysulfides via covalent bonds to the cathode host material, has been demonstrated to be extremely efficient in suppressing the shuttle effect. Various organic moieties containing functional groups (e. g., allyl, thiol, or cyano) have been copolymerized with sulfur through inverse vulcanization to form the organosulfur polymers,^[4]^ along with the improvement of cycling stability. However, the poor conductivity of most organosulfur compounds has been the main drawback that hampers their advance towards practical use. As a result, conjugated polymers emerge as promising candidates due to their significantly improved conductivity when compared with small organic molecules and non‐conjugated polymers, as well as their ability to form chemical bonds with sulfur species.^[5]^

To gain insight into the structure of the organosulfur polymer, a series of ex‐situ measurements have been combined, such as X‐ray photoelectron spectroscopy (XPS), nuclear magnetic resonance (NMR) spectroscopy, and electron paramagnetic resonance (EPR) analysis,^[6]^ and confirmed the formation of covalent bonds between sulfur moieties and polymer backbones. However, more direct information based on operando analysis is still missing, which is essential to pursuing a comprehensive view of the redox mechanism and structure evolution of the organosulfur polymer during the charge‐discharge process, especially the formation of polysulfides and the reversibility of covalent bonding of sulfur species to the polymer. Therefore, developing a highly conductive, flexible, and free‐standing organosulfur cathode for operando analysis under realistic reaction conditions is of great importance.

Normally, chemical polymerization is applied for the synthesis of functional conducting polymers and then followed by the traditional fabrication of electrodes containing the binder and additive carbon, the excitation signals of which will disturb the operando analysis of the cathode. In addition, the chemical‐polymerization method often suffers from poor control of the morphology of the resulting materials. Thus, it is quite a challenge to produce well‐designed electrodes with optimized conductivity, diffusion efficiency, and so on. Therefore, some efforts have been devoted to taking advantage of the electro‐polymerizable properties of conducting polymers to construct free‐standing cathodes in situ.^[7]^ As is well known, electro‐polymerization of conducting monomers on a flexible conducting substrate has been commonly used to design and fabricate functional electronic devices, owing to their low equipment cost, facile operation, and low‐temperature process advantages. Meanwhile, Ni foam (NF), a low‐cost commercial material, has been widely used as a substrate and support for electrode materials owing to its high electronic conductivity, desirable 3D open‐pore structure, and high specific surface area.^[8]^ In addition, the porous structure can decrease the loss of X‐rays for the operando analysis and imaging.^[9]^

**Scheme 1:**
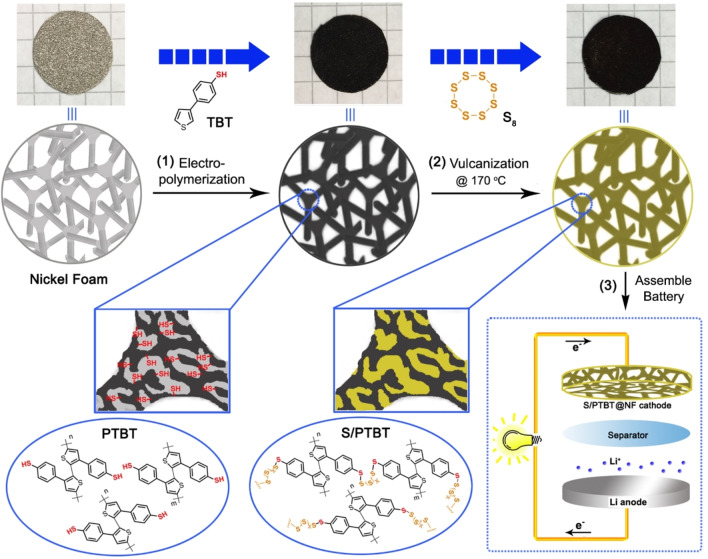
Fabrication route of binder‐ and carbon additive‐free S/PTBT@NF cathode. Schematic illustration describing the fabrication procedure of sulfur crosslinked to a PTBT cathode through a combined electrochemical and vulcanization strategy in situ: (1) creation of the PTBT frameworks on porous NF by electro‐polymerization method; (2) crosslink of ring‐opening sulfur through vulcanization; and (3) assembly of the Li‐S batteries by using the newly gained cathode material.

Thus, in the present work, we take advantage of the electro‐polymerization strategy to construct a free‐standing cathode on NF in situ. Combined with the electropolymerizable thiophene molecule as the main chain and the benzenethiol containing thiol group as the lateral chain, 4‐(thiophene‐3‐yl)benzenethiol (TBT) was synthesized. The corresponding polymer with many flexible and active sulfur binding sites was applied as the framework to fabricate a novel binder‐ and carbon additive‐free cathode of Li‐S battery by two steps as shown in Scheme 1. In the first step, TBT monomers were electro‐polymerized on the surface of flexible NF to form the porous and conducting poly(4‐(thiophene‐3‐yl)benzenethiol) (PTBT) frameworks with many lateral chains of thiol groups, which could serve as chemical binding sites for polysulfides. In the second step, this framework was thoroughly combined with sulfur molecules through vulcanization to obtain an organosulfur S/PTBT@NF cathode, which could be used to assemble Li‐S batteries without any further modification. The loose structure of PTBT frameworks and the porous structure of NF are beneficial for the penetration and diffusion of electrolytes; meanwhile, the interconnected conjugate structure can be used as the transfer channels of electrons. Most importantly, this free‐standing cathode can be applied as a model system for an operando study of the batteries. In this work, operando X‐ray imaging analysis was applied to detect the sulfur storage manner of the organosulfur cathode during the charge‐discharge process. This strategy does not only take advantage of the chemical confinement of the conducting polymer to improve the performance of cathodes but also simplify the fabrication process of electrode materials for Li‐S batteries. Moreover, owing to the electro‐deposition method, electrodes with different sizes can be easily obtained, as well as substituting NF with other flexible, cheaper, and higher‐conductive substrates. We believe this facile fabrication method of free‐standing organosulfur cathodes has great potential in the exploitation of highly efficient Li‐S batteries in the future.

## Results and Discussion

**Figure 1:**
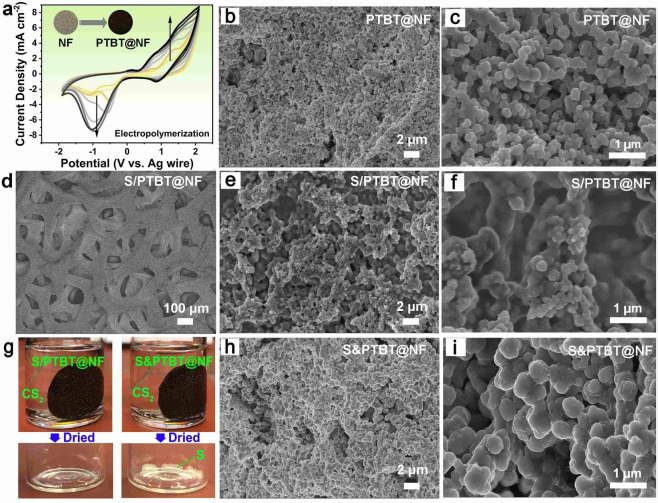
(a) Repeated potential scan electro‐polymerization of TBT in 0.1 m TBAPF_6_/ACN at 100 mV s^−1^ on NF. (b,c) SEM images of PTBT@NF and (d–f) S/PTBT@NF with low and high magnification. (g) Pictures of S/PTBT@NF (left) and S&PTBT@NF (right) electrode immersed in CS_2_ and the corresponding product after drying of CS_2_. (h,i) SEM images of S&PTBT@NF with low and high magnification.

To fabricate the PTBT through electro‐polymerization, the thiol‐containing monomer TBT was firstly synthesized as shown in Scheme S1, and the detailed experimental conditions and characterization results are shown in the Supporting Information (S1). The final product of TBT is a light‐yellow powder as shown in Figure S1. For the fabrication of the S/PTBT@NF cathode, the flexible nickel foam was used as the substrate for electro‐polymerization of TBT as well as the current collector of the cathode. To be more specific, the electro‐polymerization of the TBT monomers was carried out in acetonitrile solution to form PTBT on the surface of NF, which leads to the formation of PTBT@NF frameworks. Such frameworks not only provide continuous pathways for electron transport due to the internal conjugated main‐chain but also furnish plenty of lateral thiol groups working as active bonding sites, which can fix sulfur and polysulfide through the formation of S−S bonds for their chemical confinement. Figure 1a described the representative electrochemical growth processes of TBT on NF by cyclic voltammetry (CV) for oxidative electro‐polymerization in an acetonitrile (ACN) solution containing 2 mg mL^−1^ TBT monomer and 0.1 m tetrabutylammonium hexafluorophosphate (TBAPF_6_) as the electrolyte, revealing the electroactivity of TBT and the formation of PTBT on the surface of NF. During this process, a dark red‐brown PTBT film forming on the surface of NF can be observed (inset of Figure 1a) with the increasing of peak currents in CV curves. The first cycle of the CV curve for the electrochemical property of TBT is shown in Figure S2. After seven circles, the CV curves becoming stable indicates the maximum deposition of PTBT with a weight of about 1.0 mg cm^−2^ after rinsing by ACN and drying at room temperature. With prolonging the electro‐polymerization time, the PTBT film became thicker and looser, from which the outer layer was likely to fall off uniformly. Scanning electron microscopy (SEM) images have been taken to investigate the microstructures of the obtained PTBT@NF sample. As shown in Figure 1b,c, the PTBT@NF electrode exhibits a loose structure, where the obtained PTBT layer is composed of some integrated particles with the size of 100–300 nm. The Brunauer‐Emmett‐Teller (BET) surface area was recorded as 6.9 m^2^ g^−1^ with a range of pore dimensions (3–10 nm) (Figure S3). It is necessary to stress that these particles are homogeneously coated on the NF, which can provide a favorable pathway for charge transfer. In addition, the loose structure of the polymer can facilitate the quick diffusion of electrolytes.

To produce the S/PTBT@NF cathode with the linear polysulfane along the thiol surface, a two‐step vulcanization process was carried out in a sealed vessel: sulfur was first embedded into the PTBT framework at a low‐temperature step at 150 °C, followed by further heating to 170 °C leading to ring‐opening radical polymerization of elemental sulfur with the thiol groups of PTBT.^[5b]^ After rinsing with CS_2_ to remove the physical‐adsorbed sulfur and drying at room temperature, the free‐standing S/PTBT@NF cathode can be obtained and employed to assemble the Li‐S battery without any further modification. A control cathode without heating treatment to sulfur, namely S&PTBT@NF, was employed for comparison. It is instructive that this strategy can be used as an alternative method for the fabrication of organosulfur cathode electrodes without binder and carbon additives. Our design and synthesis of the new conducting thiol‐polymer PTBT using the electropolymerization method could pave the way for the exploration of advanced free‐standing cathode materials with the function of covalent fixing of sulfur species.

In order to vulcanize the PTBT@NF thoroughly, a certain amount of sulfur was dissolved in the CS_2_ solution to infiltrate the frameworks in advance. Then the vulcanization process was carried out according to the methods mentioned above. After vulcanization, the S/PTBT@NF electrode with a dark‐brown color was obtained as shown in Scheme 1. The SEM images in Figure 1d–f show a smooth surface and disappearance of most of the pores. In addition, the cross‐section SEM image in Figure S4 also shows a similar morphology with a thickness of about 17 μm. The S/PTBT@NF electrode is stable in CS_2_ solvent without dissolution of sulfur species (Figure 1g), which indicates that the sulfur molecules should be fixed to PTBT through covalent bonds with thiol groups in the polymer. The elemental content and corresponding element maps of PTBT@NF electrodes before and after vulcanization are shown in Figure S5. The element weight percent of S is increased from 14.7 to 66.8 % after vulcanization, denoting that plenty of sulfur was loaded in the way of chemical bonding with PTBT. The sulfur loading in S/PTBT@NF is about 1.6 mg cm^−2^. Different from the morphology of PTBT@NF, the physically adsorbed sulfur sample (named S&PTBT@NF) was composed of some larger particles with the size of around 500 nm and most of the pores are still present (Figure 1h,i). The growing size of the particles indicates that the sulfur was prone to distribute and coat on the surface of PTBT, which ensures the full reaction between sulfur and PTBT during vulcanization. In addition, the S&PTBT@NF electrodes are light‐yellow in color, indicating the surface precipitation of sulfur, which can be defined as physical adsorption due to the fact that the sulfur in S&PTBT@NF can be totally removed by CS_2_, and the weight of the eluted sulfur from the electrode is almost the same as the amount of initial addition (Figure 1g).

**Figure 2:**
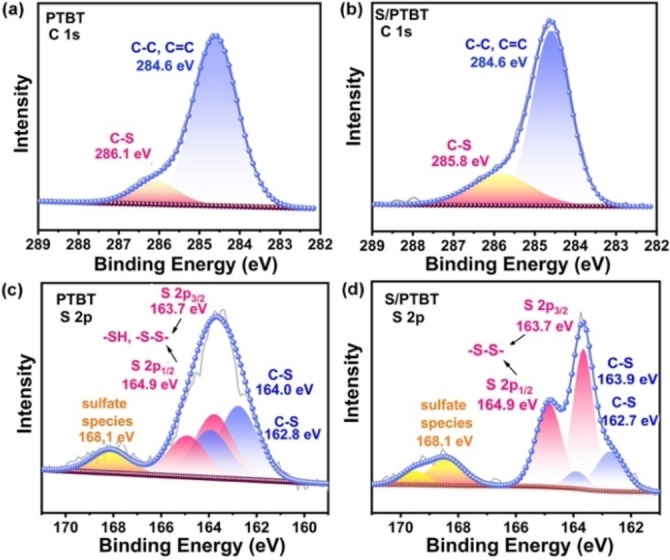
(a,b) C 1s and (c,d) S 2p XPS spectra of PTBT and S/PTBT samples.

To deeply investigate the chemical‐bonding interaction between sulfur and PTBT in the S/PTBT electrodes, molecular and structural characteristics of the vulcanized PTBT were examined by means of different measurements. Firstly, XPS measurements were carried out to investigate the variation of chemical states and the elemental composition in the vulcanized PTBT. As shown in high‐resolution C 1s XPS spectra of Figure 2a,b, both samples of PTBT and S/PTBT show two peaks: one peak at 284.6 eV belongs to the C−C/C=C bond of benzol and thiophene skeleton, and the other peak is the C−S bond of thiophene ring and thiol group, respectively.^[5a]^ Note that S/PTBT possesses lower binding energy of 285.8 eV for C−S bond than PTBT (286.1 eV) as well as a little increased content, indicating the stronger interaction between the linear sulfur and C−S bond of thiol after vulcanization. The corresponding high‐resolution S 2p spectra are presented in Figure 2c,d, where three species of sulfur including C−S, −SH, or −S−S− and the sulfate species can be observed at the peaks of 162.7, 163.7, and 168.1 eV with their satellite peaks.^[10]^ It is obvious that after vulcanization, the content of −S−S− bond increased distinctly; these increased S−S bonds possess lower binding energy than that of pure sulfur S_8_ (164.0 eV),^[11]^ which indicates that the added sulfur molecules in S/PTBT were ring‐opened and covalently bonded to the PTBT through the formation of S−S bonds with the thiol groups. Besides, the sulfate species at 168.1 eV are attributed to the doping process of the thiophene ring during the electro‐polymerization to ensure the conductivity, which is commonly observed in the polythiophene or poly(3,4‐ethylene‐dioxythiophene)(PEDOT).^[12]^

**Figure 3:**
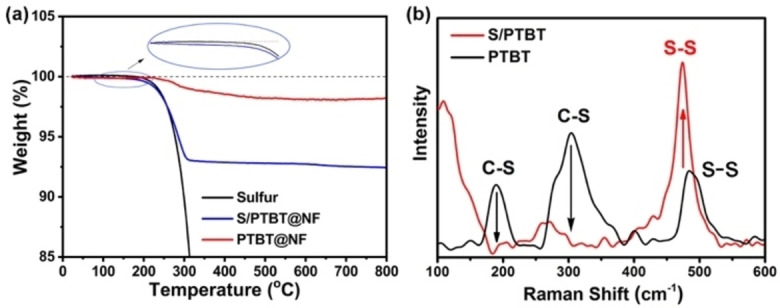
(a) TGA analysis and (b) Raman spectra of PTBT and S/PTBT samples.

**Figure 4:**
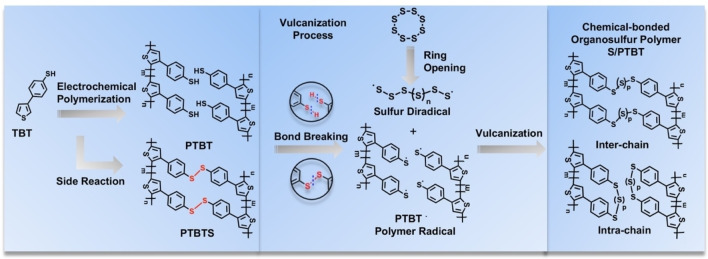
Illustration of the molecular structure evolution during the whole fabrication process.

Then, the thermogravimetric analysis (TGA) curves of different samples in argon are displayed in Figure 3a. The negligible weight loss of PTBT@NF below 200 °C indicates that the PTBT is thermally stable under the condition of vulcanization. Moreover, S/PTBT@NF exhibits a lower sublimation temperature (inset of Figure 3a) and a gentler slope of thermal decomposition than the pure sulfur S_8_. The low sublimation temperature and slow loss process are related to the release of sulfur covalently bonded to the PTBT frameworks.^[4b]^ These results support the covalent attachment of sulfur to the PTBT frameworks during the functional fabrication of cathodes in situ for Li‐S batteries. Furthermore, the Raman spectra of the PTBT and S/PTBT samples are shown in Figure 3b. After vulcanization, the characteristic peak of S−S bonds at 474 cm^−1^ was present with a remarkable intensity in S/PTBT,[^4b^, ^4g^, ^13^] which indicates successful chemical bonding of sulfur to the polymer frameworks. The intensities of the peaks in PTBT became relatively weak, including the C−S peaks at 182 and 308 cm^−1^, which may be due to the suppression by the fed sulfur.^[14]^ Notably, the last peak of PTBT at 480 cm^−1^ should belong to the disulfides S−S bonds formed between inter‐chain thiol groups (−SH⋅⋅⋅SH−) of PTBT molecules as a side reaction during or after the electrochemical polymerization, which has also been reported in the literature.^[15]^ To verify the phenomenon, we have also measured the Raman spectra of a TBT monomer, which is shown in Figure S6. The signal around 2560 cm^−1^ belongs to the S−H stretching vibration mode of the thiol group in the monomer. After electrochemical polymerization, this signal disappears, and a peak emerges at 480 cm^−1^ that indicates the formation of disulfide S−S bonds in the PTBT polymer. The shift of the S−S peak position in the Raman spectra of S/PTBT (Figure 3b) shows that the S−S bonds in PTBT (C−S−S−C) are different from the emerged S−S bonds after vulcanization. This fact strongly suggests that the disulfide S−S bonds in PTBT were broken and then reacted with sulfur radicals to form longer polysulfide chains (.S−S−S−S.) during the vulcanization process. Taking these results into account, we have proposed the rational molecular structure evolution during the whole fabrication process (Figure 4). On the first step of electrochemical polymerization, the monomer TBT was polymerized to form the PTBT polymer, and a side reaction happened between the inter‐chain thiol group leading to the disulfide polymer named PTBTS. Next on the vulcanization process, the S_8_ monomer undergoes ring‐opening polymerization (ROP) into linear polysulfide with diradical chain ends.^[4a]^ Meanwhile, the S−H bond of PTBT polymer breaks to form the polymer radical (PTBT⋅). The lost two hydrogen atoms react with the sulfur atom during the vulcanization, releasing H_2_S gas as a bi‐product, which has already been confirmed by experiments.^[16]^ For the PTBTS polymer, the cleavage of disulfide S−S bonds leads to the formation of the same polymer radical (PTBT⋅), which then reacts with the sulfur diradical. Note that no H_2_S gas is produced in this route. Finally, the chemical‐bonded organosulfur polymer S/PTBT is formed. According to the findings in our recent work, in which we have investigated the initial structure of the cathode using a combination of electronic‐structure theory and statistical mechanics,^[17]^ we have proposed two possible structures for the S/PTBT polymer, named inter‐chain and intra‐chain, which can be generally distinguished by the way in which the polysulfide chains bind to the TBT units. From these two possible structures, our results showed that pentasulfide (p=5) inter‐chain crosslinks are dominant after vulcanization.^[17]^

**Figure 5:**
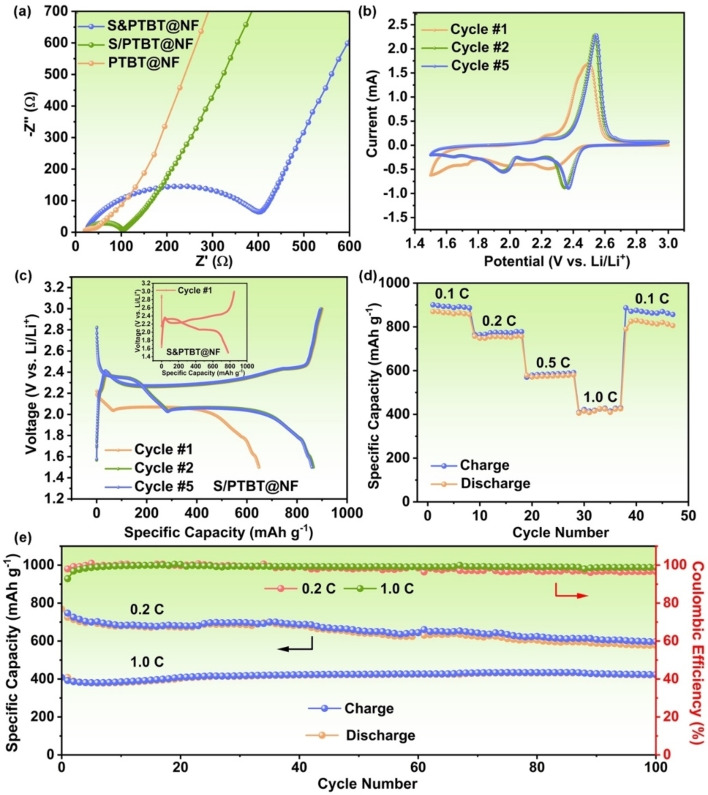
(a) Nyquist plots of PTBT@NF, S/PTBT@NF, and S&PTBT@NF cathode. (b) CV of S/PTBT@NF cathode at a scan rate of 0.1 mV s^−1^. (c) Representative galvanostatic discharge/charge voltage profiles of the S/PTBT@NF cathode for the 1st, 2nd, and 5th cycles at 0.1 C. Discharge/charge voltage profiles of the S&PTBT@NF cathode at the first cycle are also shown as inset. (d) Rate capabilities of the S/PTBT@NF cathode in 1.5–3.0 V at various current densities. (e) Discharge/charge capacities and coulombic efficiencies of the S/PTBT@NF cathodes for 100 cycles (after the first cycle) at the different C rates.

**Figure 6:**
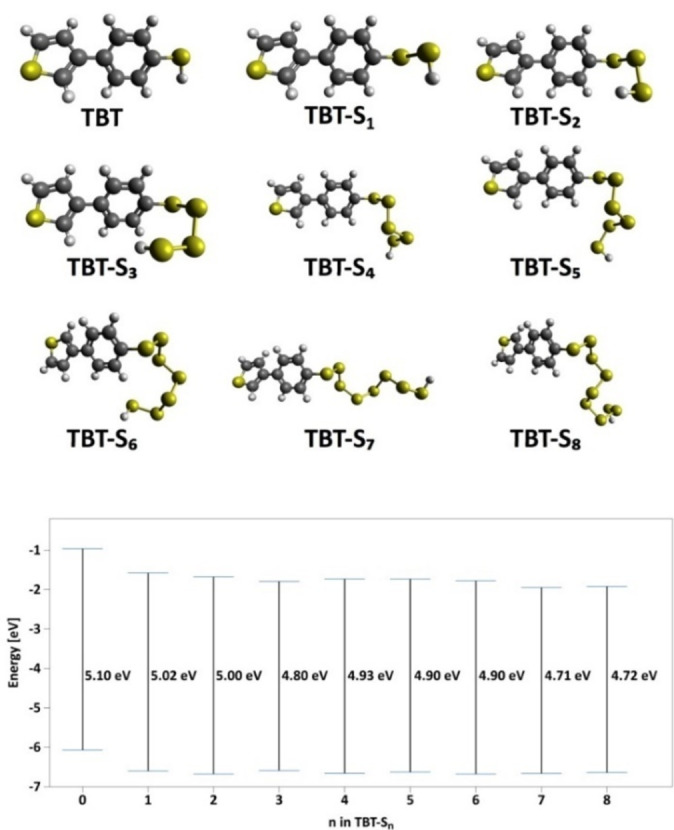
PBE0+vdW calculations of the HOMO‐LUMO gap of TBT and TBT‐Sn molecules. All the model structures shown correspond to the most stable configuration after optimization of the forces as described in the Experimental Section. Carbon (C), hydrogen (H), and sulfur (S) elements are displayed as spheres in grey, white, and yellow, respectively.

The S/PTBT@NF cathodes without carbon additives and binder are directly applied in the Li‐S coin cells. To investigate the effect of interaction between sulfur and PTBT on the conductivity of the cathode, the analysis of the electrochemical impedance spectroscopy (EIS) was first evaluated for Li‐S batteries. Figure 5a shows the Nyquist plots of the pristine PTBT@NF, the chemical bonded S/PTBT@NF, and the control sample S&PTBT@NF cathode. All three electrodes show a semicircle in the high‐frequency region associated with the charge‐transfer resistance (*R*_ct_). It can be observed that the pristine PTBT@NF shows the smallest *R*_ct_ of around 20 Ω, due to the good conductivity of PTBT polymer. After sulfur embedding, the *R*_ct_ of the control cathode S&PTBT@NF increases significantly (∼400 Ω), most likely due to the insulating effect of elemental sulfur. Notably, after vulcanization, the *R*_ct_ of the S/PTBT@NF cathode decreased remarkably to around 75 Ω, indicating that covalent crosslinking of sulfur to PTBT leads to enhanced electron conduction in the cathode. This resistance value is comparable to those reported for organosulfur polymers with conductive carbon additives, which are summarized in Table S1, and reflects the good conductivity of the S/PTBT@NF cathode in the absence of additional conducting agents. The decrease of the resistance to electronic conduction in the S/PTBT@NF cathode occurring upon vulcanization can be related to a change in its electronic structure. Density‐functional theory (DFT) calculations were carried out to calculate the energy bandgap of a TBT monomer upon attachment of sulfur chains with different numbers of sulfur atoms. Figure 6 shows the optimized structures and the highest occupied molecular orbital (HOMO)‐lowest unoccupied molecular orbital (LUMO) gap of TBT monomers after attachment of *n*=2–8 sulfur atoms, calculated with the PBE0 exchange‐correlation functional^[18]^ plus van der Waals interactions using the Tkatchenko‐Scheffler method (PBE0+vdW).^[19]^ The results show a reduction of the HOMO‐LUMO gap upon extension of the sulfur chain, gradually decreasing from 5.10 eV for TBT to 4.72 eV after attachment of an S_8_ chain. These results suggest that covalently bonded sulfur could indeed enhance the conducting properties of the PTBT cathode. In addition, it is important to note that the conducting properties of such a polymeric cathode can be further tuned by many synthetic design parameters such as polydispersity, compositional fluctuations, and morphological properties.^[20]^ The electrochemical performance of S/PTBT@NF was further studied: Figure 5b exhibits the CV of the S/PTBT@NF cathode, which is similar to a typical Li‐S redox curve. But the remarkable thing is at the first cycle, only one broad and weak reduction peak was observed, then two obvious reduction peaks emerged from the following cycles. The repetitive galvanostatic discharge/charge cycling between 1.5 and 3.0 V at 0.1 C (1 C=1675 mA g^−1^) was displayed in Figure 5c. It is notable that there is only one distinct plateau at 2.07 V (vs. Li/Li^+^) during the first discharge process, which is consistent with the CV result, while two plateaus at 2.33 and 2.06 V are present for the control S&PTBT@NF cathode (inset of Figure 5c). According to the previous studies, the two voltage plateaus are attributed to the reduction of S_8_ to high‐order polysulfides Li_2_S_*x*_ (*x*=4–8) at 2.35 V and further to low‐order polysulfides Li_2_S_2_ and Li_2_S at 2.07 V, respectively.^[21]^ This result further confirmed that most of the sulfur in the S/PTBT@NF cathode were ring‐opened and covalently bonded to the PTBT through forming S−S bonds. This phenomenon is similar to those organosulfur cathodes, for which the ring‐opening plateaus in the first discharge process are disappeared.^[4b]^ However, this discharge plateau has appeared and is stable at 2.35 V in the following discharge/charge cycles of S/PTBT@NF, which suggests the formation of S_8_ molecules after the scission of crosslinked sulfur side chains and regeneration of S−S bonds with cycling. Moreover, the S/PTBT@NF exhibits a low initial discharge capacity of around 650 mAh g^−1^ and a stable capacity of around 860 mAh g^−1^ for the following cycles. The reversible discharge/charge capacities at various C‐rate are exhibited in Figure 5d. Although the C‐rate capability is inferior to the similar sulfur‐containing cathodes, S/PTBT@NF without the assistance of super P carbon delivered a considerable reversible capacity of around 870 mAh g^−1^ at 0.1 C, 756 (0.2 C), 576 (0.5 C), 417 (1.0 C), and back to a reversible capacity of 820 mAh g^−1^ at 0.1 C. Meanwhile, the S/PTBT@NF exhibited good capacity retention, in which the capacity is conserved at around 600 mAh g^−1^ for 0.2 C (≈80 %) and 400 mAh g^−1^ for 1.0 C (≈96 %) after 100 cycles, with the coulombic efficiencies of 97 and 99 %, respectively, in Figure 5e. It is notable that when cycling at 0.2 C, the achieved capacity is higher and the coulombic efficiency declines along with cycling, which can be ascribed to the structural deformation of the electrode materials induced by the larger storage of Li ions. In contrast, when cycling at 1.0 C, the lithiation capacity of the electrode materials is limited with minimal structural deformation, and therefore, the cycling stability is enhanced, and the coulombic efficiency does not decay that as rapidly as during cycling at a low rate (0.2 C). Such phenomenon has been frequently observed in alloy‐Si^[22]^ or conversion‐type electrode^[23]^ materials for Li‐ion batteries that undergo significant volume variations during battery charge and discharge. As a comparison, the control S&PTBT@NF, in which the loading sulfur is fixed through physical adsorption, showed a markedly low discharge capacity of below 400 mAh g^−1^ for 0.2 C, while maintaining around 83 % capacity (compared with the first discharge capacity) after 100 cycles with a coulombic efficiency of below 90 % throughout, as shown in Figure S7. The significantly improved capacity and coulombic efficiency of S/PTBT@NF when compared to that of S&PTBT@NF should be attributed to the chemical‐bonding interaction between sulfur and PTBT. The long‐term discharge‐charge cycling performance in Figure S8 shows that the capacity of the S/PTBT@NF cathode declined slightly with increasing cycle number at a fading rate of 0.178 and 0.387 % per cycle for 200 and 300 cycles. Furthermore, the morphology of the S/PTBT@NF electrode after 100 cycles looks similar to that of the original S/PTBT@NF electrode as shown in Figure S9, which indicates that most of the attached sulfur was confined in the cathode during fully repetitive lithiation and delithiation reactions. In summary, these coin‐cell battery performances have verified the S/PTBT@NF cathode can work efficiently in the absence of additional conducting agents.

**Figure 7:**
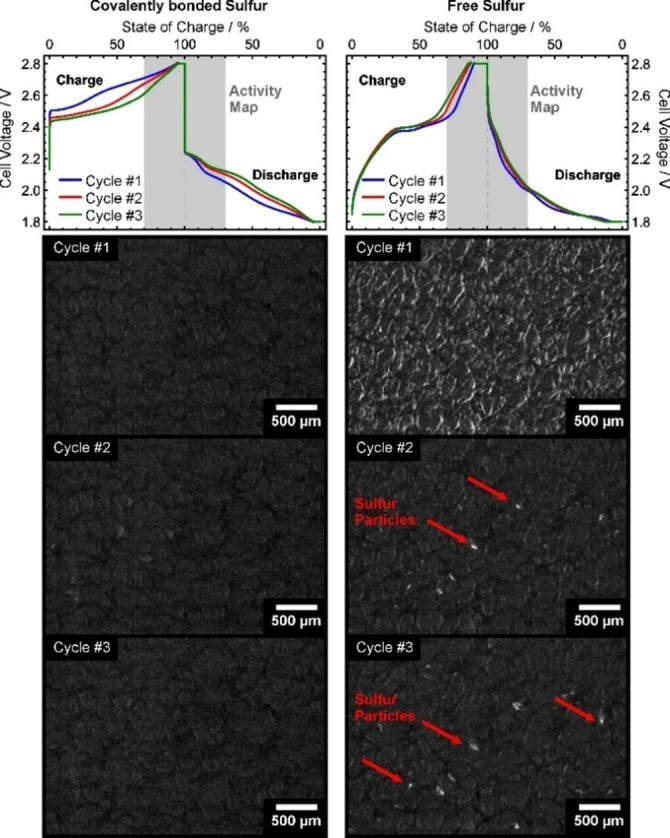
Results of the operando X‐ray imaging study for covalently bonded sulfur to the PTBT‐cathode and free sulfur on the PTBT cathode. The activity map was created from the X‐ray images around the charged state (±30 % state‐of‐charge).

As a proof of concept, the porous, binder‐free, and free‐standing cathode without additional carbon additives can work as the model system for operando analysis. X‐ray imaging of the operating Li/S cell is a suitable tool to test the effect of the polymer. If sulfur is covalently bonded to the polymer, no macroscopic sulfur particles should appear on the cathode during charge as it was already shown for the conventional Li/S cells.^[24]^ However, if free sulfur is added to the PTBT electrode via simple drop‐casting of a sulfur‐rich CS_2_ solution, sulfur particles should form during the charge step. Figure 7 summarizes the operando X‐ray study by the device presented in Figure S12 that shows the morphological activity of the Li/S cells around the state of charge (±30 % state‐of‐charge) for both cases of sulfur admixture.

For the S/PTBT@NF cathode with covalently bonded sulfur no morphological activity can be detected, while the PTBT cathode with the free sulfur shows a clear formation of sulfur particles (marked by red arrows in Figure 7), especially in the second and third cycle. Video S1 shows the time‐dependent morphological evolution of the particles during the period of the activity map. Video S2 shows the same time interval, which is extended to the final discharge of the third cycle. Here, also the dissolution of the sulfur particles can be observed. These results clearly reveal the remarkable effect in the suppression of polysulfides shuttle via introducing covalent bonds between sulfur species and the active thiol groups, which leads to a significant improvement in the cycling performance when compared to the physically adsorbed sulfur‐based cathode. This work not only demonstrates a new fabrication strategy of the organosulfur cathodes for Li‐S batteries but also shows a way for the construction of a model cathode for the operando analysis of Li‐S batteries. The significant fixing effect of sulfur species was proven by operando X‐ray imaging in real‐time for the first time.

## Conclusion

Combined with the electro‐polymerization and vulcanization method, a porous framework based on a conducting thiol‐polymer was constructed, and the sulfur‐rich cathode from elemental sulfur without binder and carbon additives was fabricated in situ. This approach maintains the conductivity and flexibility of the polymer framework. Simultaneously, the thiol groups on its side chain can realize the covalent bonding with the long sulfur chains to further improve the performance of Li‐S batteries through the chemical confinement of polysulfide. This work not only demonstrates a new fabrication strategy of the organosulfur cathodes for Li‐S batteries but also shows a way for the construction of a model cathode for the operando analysis of Li‐S batteries. The significant fixing effect of sulfur species was proven by operando X‐ray imaging in real‐time for the first time.

## Experimental Section

### Electro‐polymerization of TBT on NF (PTBT@NF)

The commercial Ni foam (thickness: 0.5 mm) was cleaned first with acetone and soaked in 2.0 m HCl for 10 min, then washed subsequently with water and ethanol for several times, and dried in a vacuum oven at room temperature. The monomer TBT was electro‐polymerized on the Ni foam in an N_2_‐saturated acetonitrile solution containing 2 mg mL^−1^ TBT and 0.1 m TBAPF_6_ (Ttetrabutylammonium hexafluorophosphate). This process was conducted by CV in a three‐electrode system at a potential between −1.8–1.8 V (100 mV s^−1^, *n*=7) using an electrochemical workstation (GAMRY‐1100), Ag wire as the reference electrode, and platinum wire as the counter electrode. After electro‐polymerization, the electrode was rinsed with acetonitrile several times and then dried in a vacuum oven at room temperature. Finally, a red‐brown electrode (PTBT@NF) was obtained, and the mass loading of PTBT is around 1.0 mg cm^−2^.

### Preparation of S/PTBT@NF and S&PTBT@NF cathodes

In advance, a certain weight of sulfur was dissolved in 100 μL CS_2_. Then the sulfur was mixed with PTBT@NF through dropping and quickly drying the S/CS_2_ solution on a hot stage at 60 °C to obtain the control S&PTBT@NF electrode. After that, the S&PTBT@NF was heated at 150 °C for 1 h under an argon atmosphere, facilitating the melt infiltration of sulfur into the polymer framework. Then followed by further heating at 170 °C for 8 h to form the S/PTBT@NF electrode. Finally, the S/PTBT@NF was rinsed with CS_2_ to wash away the physical‐adsorbed sulfur. The loading amount of sulfur in S/PTBT@NF is around 1.6 mg cm^−2^, which was calculated through the weight variation of S/PTBT@NF and PTBT@NF. The equivalent sulfur in control S&PTBT@NF electrodes was predetermined by controlling the drop volume of the S/CS_2_ solution. The resultant S&PTBT@NF and S/PTBT@NF electrodes exhibited a light‐yellow and dark‐brown color, respectively.

### Materials characterizations

The morphologies and structures of the samples were examined by SEM (LEO 1530) with an energy‐dispersive X‐ray spectroscopy (EDX) attachment (Zeiss). XPS (Thermo Scientific, Escalab 250Xi) was employed to analyze the composition of the samples. TGA was performed in a temperature range of 25–800 °C with a heating rate of 10 °C min^−1^ under an argon atmosphere. Raman spectra were measured using a Horiba LabRAM HR 800 Raman spectroscopy. Nitrogen adsorption experiments were performed with a Quantachrome Autosorb‐1 at liquid nitrogen temperature, and data analysis was performed by Quantachrome software. The specific surface area was calculated using the BET equation. Pore size distribution was determined by Barrett‐Joyner‐Halenda (BJH) method.

### Battery tests

The Li‐S batteries were assembled in an Ar‐filled glovebox with the concentration of moisture and oxygen below 1.0 ppm. Coin type (CR 2032) cells were fabricated by assembling an S/PTBT@NF or S&PTBT@NF cathode, a Celgard 2500 diaphragm separator, and a lithium foil anode with 30 μL electrolyte [electrolyte/sulfur (E/S) ratio≈18.7]. The electrolyte was prepared by dissolving 1 m lithium bis(trifluoromethane)sulfonamide (LiTFSI) and 0.1 m lithium nitride (LiNO_3_) in a mixture solution of 1,3‐dioxolane and 1,2‐dimethoxyethane (DOL/DME; 1 : 1 *v*/*v*). The galvanostatic charge/discharge tests were performed using a Bio‐Logic VMP3 electrochemical workstation at different current densities within a cutoff voltage window of 1.5–3.0 V. The specific capacity is calculated based on the mass of sulfur. EIS was also carried out by applying an AC voltage with a 5 mV amplitude in a frequency of 0.01 to 100 kHz at open‐circuit potential.

### Operando X‐ray imaging

The operando cell design used for this study is shown in the Supporting Information. The inner geometry of the cell is the same as for CR2032 standard coin cells. The cell was assembled with two lithium chips to achieve a significant electrode pressure. Two Celgard 2500 separators made of porous polypropylene were used to minimize the risk for a short circuit due to the sharp edges of the nickel foam. Subsequently, the respective PTBT cathode either with covalently bonded or free sulfur, with 1.5 mg of sulfur each, was placed in the operando cell. Finally, 50 μL of the electrolyte (DOL/DME 1 : 1 *v*/*v*, 1 m LiTFSI, 0.1 m LiNO_3_) was added on the porous Ni foam. After applying a vacuum for less than one minute to achieve a complete filling of the cell by the electrolyte, the additional electrolyte was added onto the Ni‐foam cathode until a proper wetting was obtained. This results in 136.6 mg and 90.3 mg of electrolyte in the operando cell for the covalently bonded and free sulfur cathode, respectively. The open‐circuit potential for each electrochemical cell was measured immediately after assembling and was 2.939 and 2.623 V for covalently bonded and free sulfur, respectively. The cells were cycled with a C‐rate of 0.1 C in the voltage window that ranged from 1.8 to 2.8 V. EIS was performed at the end of each charge and discharge step. The X‐ray radiography images were recorded by using a laboratory CT setup (Supporting Information). The applied voltage of the tungsten source was 100 keV and the current was set to 100 μA. The images were detected with a flat panel argon detector every 20 s with a resolution of approximately 10 μm per pixel. The images were post‐processed with the freely available open‐source software ImageJ. The images were cropped to the region of interest after dark field correction and flat field division. An activity map was created for the first three states of charge to examine the appearance of sulfur crystals in the cell. For this, the standard deviation of all X‐ray images was taken that is 30 % state‐of‐charge away from the final charge state. Therefore, bright areas show regions with high activity while darker areas represent fields with low morphological activity. This analysis allows for the detection of particle formation despite the high attenuation of nickel for X‐rays. In addition, two videos of the 3rd cycle of the free sulfur cathode were created that clearly show the formation and dissolution of sulfur particles around the charged state. Here, a background image of the Ni foam was created by calculating the mean value image during a period of low activity at the beginning of the charge. This image was subsequently subtracted from the image stack to achieve a better contrast with respect to the formed sulfur particles. Video S1 shows the time period where the activity map of the 3rd cycle of the free sulfur cathode was created from. Video S2 shows the same period but is extended to the end of discharge.

### Theoretical calculations

The computational details of the theoretical calculations can be found in section S2 of the Supporting Information.

## Conflict of interest

The authors declare no conflict of interest.

1

## Supporting information

As a service to our authors and readers, this journal provides supporting information supplied by the authors. Such materials are peer reviewed and may be re‐organized for online delivery, but are not copy‐edited or typeset. Technical support issues arising from supporting information (other than missing files) should be addressed to the authors.

Supporting Information

Supporting Information

Supporting Information

## Data Availability

The data that support the findings of this study are available from the corresponding author upon reasonable request.

## References

[1] A. Manthiram , Y. Fu , Y. Su , Acc. Chem. Res. 2013, 46,1125–1134. 23095063 10.1021/ar300179v

[2] H. Peng , J. Huang , X. Cheng , Q. Zhang , Adv. Energy Mater. 2017, 7, 1700260.

[3a] M. Shi , S. Zhang , Y. Jiang , Z. Jiang , L. Zhang , J. Chang , T. Wei , Z. Fan , Nano-Micro Lett. 2020, 12, 146; 10.1007/s40820-020-00477-3 PMC7770931 34138132

[3b] L. Xue , L. Zeng , W. Kang , H. Chen , Y. Hu , Y. Li , W. Chen , T. Lei , Y. Yan , C. Yang , A. Hu , X. Wang , J. Xiong , C. Zhang , Adv. Energy Mater. 2021, 11, 2100420;

[3c] H. Zhang , L Yang , P. Zhang , C. Lu , D. Sha , B. Yan , W. He , M. Zhou , W. Zhang , L. Pan , Z. Sun , Adv. Mater. 2021, 33, 2008447; 10.1002/adma.202008447 33864408

[3d] J. Qian , Y. Xing , Y. Yang , Y. Li , K. Yu , W. Li , T. Zhao , Y. Ye , L. Li , F. Wu , R. Chen , Adv. Mater. 2021, 33, 2100810; 10.1002/adma.202100810 33987896

[3e] B. Liu , R. Bo , M. Taheri , I. Bernardo , N. Motta , H. Chen , T. Tsuzuki , G. Yu , A. Tricoli , Nano Lett. 2019, 19, 4391–4399. 31246030 10.1021/acs.nanolett.9b01033

[4a] W. Chung , J. Griebel , E. Kim , H. Yoon , A. Simmonds , H. Ji , P. Dirlam , R. Glass , J. Wie , N. Nguyen , B. Guralnick , J. Park , Á. Somogyi , P. Theato , M. Mackay , Y. Sung , K. Char , J. Pyun , Nat. Chem. 2013, 5, 518–524; 23695634 10.1038/nchem.1624

[4b] H. Kim , J. Lee , H. Ahn , O. Kim , M. Park , Nat. Commun. 2015, 6, 7278; 26065407 10.1038/ncomms8278 PMC4490390

[4c] A. Hoefling , D. Nguyen , Y. Lee , S. Song , P. Theato , Mater. Chem. Front. 2017, 1, 1818–1822;

[4d] X. Li , L. Yuan , D. Liu , Z. Li , J. Chen , K. Yuan , J. Xiang , Y. Huang , Energy Storage Mater. 2020, 26, 570–576;

[4e] R. Guan , L. Zhong , S. Wang , D. Han , M. Xiao , L. Sun , Y. Meng , ACS Appl. Mater. Interfaces 2020, 12, 8296–8305; 31985210 10.1021/acsami.9b21481

[4f] N. Xu , T. Qian , X. Liu , J. Liu , Y. Chen , C. Yan , Nano Lett. 2017, 17, 538–543; 27977209 10.1021/acs.nanolett.6b04610

[4g] S. Je , T. Hwang , S. Talapaneni , O. Buyukcakir , H. Kim , J. Yu , S. Woo , M. Jang , B. Son , A. Coskun , J. Choi , ACS Energy Lett. 2016, 1, 566–572;

[4h] H. Hu , B. Zhao , H. Cheng , S. Dai , N. Kane , Y. Yu , M. Liu , Nano Energy 2019, 57, 2211–2855.

[5a] S. Talapaneni , T. Hwang , S. Je , O. Buyukcakir , J. Choi , A. Coskun , Angew. Chem. Int. Ed. 2016, 55, 3106–3111; 10.1002/anie.201511553 26822950

[5b] S. Zeng , L. Li , L. Xie , D. Zhao , N. Wang , S. Chen , ChemSusChem 2017, 10, 3378; 28736985 10.1002/cssc.201700913

[5c] S. Zeng , L. Li , D. Zhao , J. Liu , W. Niu , N. Wang , S. Chen , J. Phys. Chem. C 2017, 121, 2495–2503;

[5d] B. Oschmann , J. Park , C. Kim , K. Char , Y. Sung , R. Zentel , Chem. Mater. 2015, 27, 7011–7017.

[6a] M. Weret , C. Kuo , T. Zeleke , T. Beyene , B. Hwang , Energy Storage Mater. 2020, 26, 483–493;

[6b] C. Huang , K. Lin , Y. Hsieh , W. Su , C. Wang , G. Brunklaus , M. Winter , J. Jiang , B. Hwang , ACS Appl. Mater. Interfaces 2021, 13, 14230–14238; 33750110 10.1021/acsami.0c22811

[6c] W. Wang , Z. Cao , G. Elia , Y. Wu , J. Ming , ACS Energy Lett. 2018, 3, 2899–2907.

[7a] T. Schoetz , C. Ponce de Leon , A. Bund , M. Ueda , Electrochem. Commun. 2018, 89, 52–56;

[7b] P. Bairagi , N. Verma , Sens. Actuators B 2019, 289, 216–225;

[7c] V. Muniraj , R. Boukherroub , M. Shelke , ACS Sustainable Chem. Eng. 2020, 8, 6433–6441.

[8] N. Chaudhari , H. Jin , B. Kim , K. Lee , Nanoscale 2017, 9, 12231–12247. 28819660 10.1039/c7nr04187j

[9] J. Weker , M. Toney , Adv. Funct. Mater. 2015, 25, 1622–1637.

[10] J. Senkevich , C. Mitchell , G. Yang , T. Lu , Langmuir 2002, 18, 1587–1594.

[11] R. Wang , J. Yang , X. Chen , Y. Zhao , F. Pan et al., Adv. Energy Mater. 2020, 10, 1903550.

[12a] A. Taouil , F. Lallemand , J. Hihn , J. Melot , V. Patissie , B. Lakard , Ultrason. Sonochem. 2011, 18, 140–148; 20493753 10.1016/j.ultsonch.2010.04.003

[12b] N. Chanunpanich , A. Ulman , A. Malagon , Y. Strzhemechny , S. Schwarz , A. Janke , T. Kratzmueller , H. Braun , Langmuir 2000, 16, 3557–3560;

[12c] E. Mitraka , M. Jafari , M. Vagin , X. Liu , M. Fahlman , T. Ederth , M. Berggren , M. Jonssona , X. Crispin , J. Mater. Chem. A 2017, 5, 4404–4412. 10.1039/c6ta10521a PMC5436492 28580144

[13a] M. Helton , P. Chen , P. Paul , Z. Tyeklar , R. Sommer , L. Zakharov , A. Rheingold , E. Solomon , K. Karlin , J. Phys. Chem. 1976, 80, 1812–1823.

[14] Y. You , W. Zeng , Y. Yin , J. Zhang , C. Yang , Y. Zhu , Y. Guo , J. Mater. Chem. A 2015, 3, 4799–4802.

[15] S. Revin , S. John , Electrochim. Acta 2011, 56, 8934–8940.

[16] P. Sang , Y. Si , Y. Fu , Chem. Commun. 2019, 55, 4857–4860. 10.1039/c9cc01495k 30951065

[17] Y. Schütze , R. Silva , J. Ning , J. Rappich , Y. Lu , V. Ruiz , A. Bande , J. Dzubiella , Phys. Chem. Chem. Phys. 2021, 23, 26709–26720. 34842867 10.1039/d1cp04550d

[18] C. Adamo , V. Barone , J. Chem. Phys. 1999, 110, 6158.

[19] A. Tkatchenko , M. Scheffler , Phys. Rev. Lett. 2009, 102, 073005. 19257665 10.1103/PhysRevLett.102.073005

[20] S. Tajik , H. Beitollahi , F. Nejad , I. Shoaie , M. Shokouhimehr , RSC Adv. 2020, 10, 37834. 35515168 10.1039/d0ra06160c PMC9057190

[21] H. Peng , J. Huang , X. Cheng , Q. Zhang , Adv. Energy Mater. 2017, 7, 1700260.

[22] Y. Xu , D. Borsa , F. Mulder , J. Electrochem. Soc. 2018, 166, A5252.

[23] T. Quan , Y. Xu , M. Tovar , N. Goubard-Bretesché , Z. Li , Z. Kochovski , H. Kirmse , K. Skrodczky , S. Mei , H. Yu , D. AbouRas , M. Wagemaker , Y. Lu , Batteries & Supercaps 2020, 3, 747.

[24a] S. Risse , C. Jafta , Y. Yang , N. Kardjilov , A. Hilger , I. Manke , M. Ballauff , Phys. Chem. Chem. Phys. 2016, 18, 10630–10636; 27035926 10.1039/c6cp01020b

[24b] Y. Yang , S. Risse , S. Mei , C. J. Jafta , Y. Lu , C. Stöcklein , N. Kardjilov , I. Manke , J. Gong , Z. Kochovski , M. Ballauff , Energy Storage Mater. 2017, 9, 96–104;

[24c] S. Risse , A. Juhl , S. Mascotto , T. Arlt , H. Markötter , A. Hilger , I. Manke , M. Fröba , J. Phys. Chem. Lett. 2020, 11, 5674–5679. 32598155 10.1021/acs.jpclett.0c01284

