# Supplementary material for: Constructing Binder‐ and Carbon Additive‐Free Organosulfur Cathodes Based on Conducting Thiol‐Polymers through Electropolymerization for Lithium‐Sulfur Batteries
Source: ChemSusChem. 2022 May 30;15(14):e202200434. doi: 10.1002/cssc.202200434 (PMC9401019; doi:10.1002/cssc.202200434)
Supplement: Supplementary file 1 — Supporting Information [file CSSC-15-0-s002.pdf]

ChemSusChem

Supporting Information

Constructing Binder- and Carbon Additive-Free Organosulfur Cathodes Based on Conducting Thiol-Polymers through Electropolymerization for Lithium-Sulfur Batteries

Jiaoyi Ning[+], Hongtao Yu[+], Shilin Mei, Yannik Schütze, Sebastian Risse, Nikolay Kardjilov, André Hilger, Ingo Manke, Annika Bande, Victor G. Ruiz, Joachim Dzubiella, Hong Meng,* and Yan Lu*© 2022 The Authors. ChemSusChem published by Wiley-VCH GmbH. This is an open access article under the terms of the Creative Commons Attribution License, which permits use, distribution and reproduction in any medium, provided the original work is properly cited.

Table S1. Summary of the reported organosulfur cathode for lithium-sulfur batteries.

Remarks	Sulfur loading (mg cm^{-2})	Composite (active material:carbon additive:binder)	Charge-transfer resistance (R_{ct}) (ohm)	Voltage (V)	First discharge capacity (mAh g^{-1})	Retention, cycles	Ref.
poly(S-r-DIB)	0.8	75:20:5		1.7-2.6	1100 (at 0.1 C)	71 %, 270	[4a]
S-TTCA-I	0.8	60:30:10		1.7-2.7	1210 (at 0.1 C)	83 %, 450	[4b]
poly(S-co-EAE)	0.75	70:15:5		1.5-2.6	650 (at 0.1 C)	64 %, 100	[4c]
STI		80:10:10	43.4	1.7-2.8	1123 (at 0.2 C)	94 %, 350	[4d]
S/P-CTF@rGO	1.5	80:10:10	27.5	1.7-2.8	1130 (at 0.5 C)	81.4 %, 500	[4e]
S-GSH	1.0	70:20:10	65	1.5-2.8	1108 (at 0.2 C)	87 %, 450	[4f]
S-BOP	0.9	60:30:10		1.7-2.7	1149 (at 0.2 C)	92.7 %, 1000	[4g]
OPNS-50<br>OPNS-72<br>OPNS-80	1.1	70:20:10	80<br>90<br>170	1.7-2.7	650 (at 1 C)<br>889 (at 1 C)<br>1100 (at 1 C)	98 %, 200<br>91 %, 620<br>72 %, 200	[4h]
S-CTF-1		60:30:10		1.7-2.7	670 (at 0.05 C)	85.8 %, 300	[5a]
cp(S-PMAT)	1.5	80:10:10	48.7	1.5-3.0	1240 (at 0.1 C)	66.9 %, 1000	[5b]
Capped CP(S3BT)/C	1.0	65:25:10	111	1.5-3.0	1362 (at 0.1 C)	75 %, 500	[5c]
(S/S-P3HT/CB	1.0	70:25:5	40	1.7-2.8	1212 (at 0.5 C)	65 %, 100	[5d]
S/PTBT	1.6	Binder and carbon additive-free	75	1.5-3.0	870 (at 0.1 C)	96 %, 100	This work

S1. Synthesis of Monomer TBT:

1. Materials

4-Bromothioanisole (97 %, Energy Chemical), bis(pinacolato)diboron (98 %, Alligator Reagent), potassium acetate (99 %, Sigma), 1,1'-Bis(diphenylphosphino) ferrocene-palladium(II)dichloride dichloromethane complex (98 %, Energy Chemical), dichloromethane (99.5 %, Aladdin), anhydrous magnesium sulfate (99 %, Aladdin), n-hexane (95 %, Aladdin), 3-bromothiophene (97%, TCI), toluene (99.5 %, Sinopharm Chemical Reagent), potassium carbonate (99.7 %, Aladdin),

tricaprylylmethylammonium chlorid (90 %, Aladdin), tetrakis (triphenylphosphine) palladium ($Pd(PPh_3)_4$) (97%, TCI)，Sodium 2-methyl-2-propanethiolate (*t*-BuSNa) (90 %, Aldrich), hydrochloric acid (37 %, Sigma-Aldrich), carbon powders (Acros), Chloroform-d (D, 99.8 % + TMS, 0.03 %, Energy Chemical) were commercially available and used as received, the 1,4-dioxane (99.5 %, Aladdin) and N,N-dimethylformamide (99.9 %, Aladdin) were dried by molecular sieves (5A, Energy Chemical).

2. Experimental Procedures

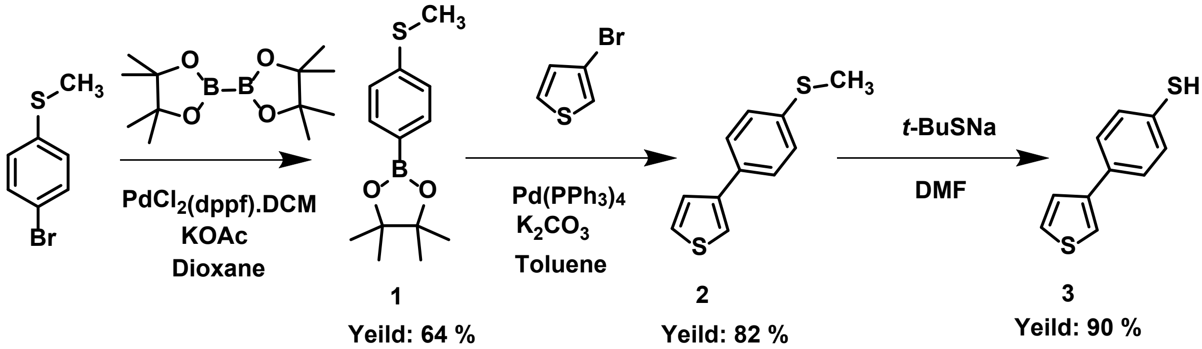

Scheme S1. Synthesis of monomer 4-(thiophene-3-yl)benzenethiol (TBT).

4,4,5,5-tetramethyl-2-(4-(methylthio)phenyl)-1,3,2-dioxaborolane (1)

A 250 mL round bottom flask were charged with 4-Bromothioanisole (10 g, 50 mmol), bis(pinacolato)diboron (16 g, 60 mmol), 200 mL dry 1,4-dioxane was added and bubbled with nitrogen for 15 minutes, then potassium acetate (6 g, 62 mmol) and 1,1'-Bis(diphenylphosphino)ferrocene-palladium(II)dichloride dichloromethane complex (0.8 g, 1mmol) were added quickly. The mixture was heated to reflux for 16 hours under the protection of nitrogen. After the reaction finished, the solvent 1,4-dioxane was moved by rotary evaporation. Then extracted with dichloromethane and water twice, the organic layer was dried with anhydrous magnesium sulfate. Finally purified by silica gel chromatography (eluent: dicholormethane and n-hexane 1:5) to give 1 as yellow oil; yield 64 %. ^{1}H NMR (300 MHz, Chloroform-d) δ 7.71 (d, J = 8.3 Hz, 2H), 7.22 (d, J = 8.3 Hz, 2H), 2.49 (s, 3H).

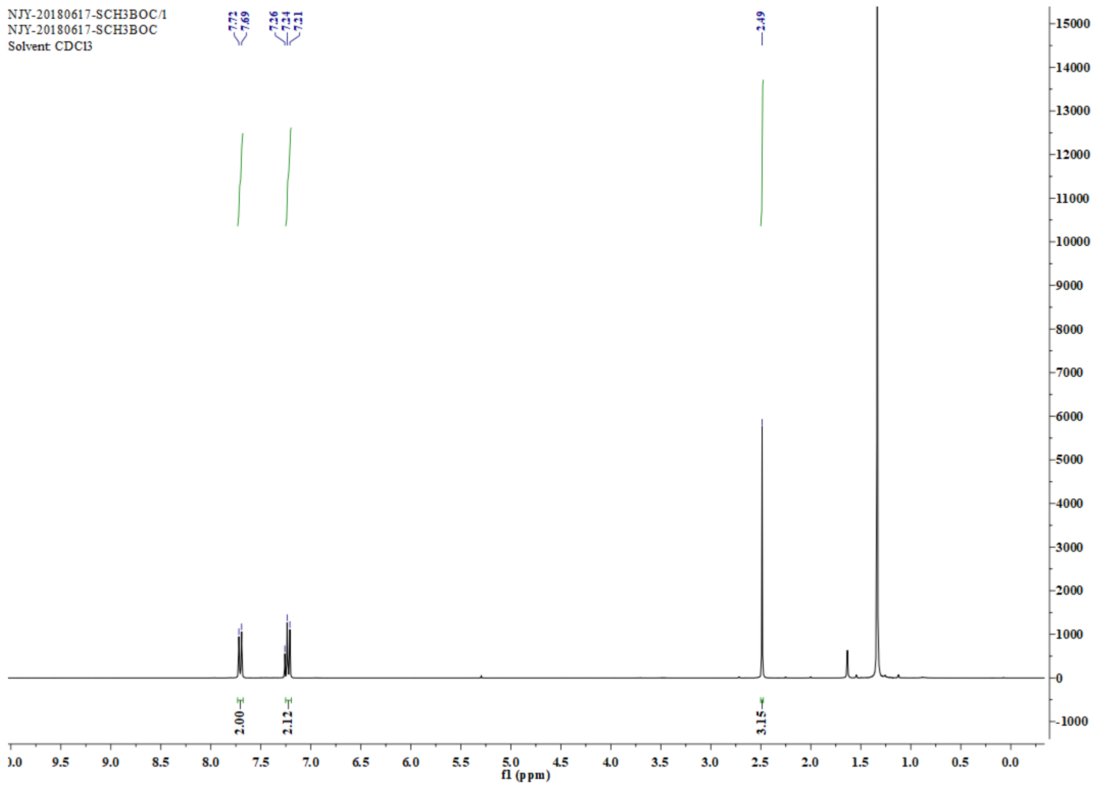

^{1}H NMR (300 MHz, $CDCl_3$) spectrum of 4,4,5,5-tetramethyl-2-(4-(methylthio)phenyl)-1,3,2-dioxaborolane.

3-(4-(methylthio)phenyl)thiophene (2) (MPT)

To a 250 mL pressure bottle, 4,4,5,5-tetramethyl-2-(4-(methylthio)phenyl)-1,3,2-dioxaborolane (**1**, 5.8 g, 23.2 mmol) and 3-bromothiophene (4.9 g, 30.16 mmol) were dissolved in 100 mL toluene. Then 35 mL 2 M potassium carbonate aqueous solution was added to the suspension followed by the addition of the phase-transfer agent tricaprylylmethylammonium chlorid. The catalyst tetrakis (triphenylphosphine) palladium ($Pd(PPh_3)_4$) (1.33 g, 1.16 mmol) were added to the mixture after bubbling with nitrogen for 15 minutes. The mixture was heated to 110°C for 24 hours. When cooling down to room temperature, the solvent toluene was moved by rotary evaporation, and future purified by silica gel chromatography (eluent: dicholormethane and n-hexane 1:10) to give 1 as white powder; yield 82%. ^{1}H NMR (300 MHz, Chloroform-d) δ 7.54 (s, 1H), 7.51 (s, 1H), 7.42 (dd, J = 2.8, 1.5 Hz, 1H), 7.38 (d, J = 2.7 Hz, 2H), 7.30 (s, 1H), 7.27 (s, 1H), 2.51 (s, 3H).

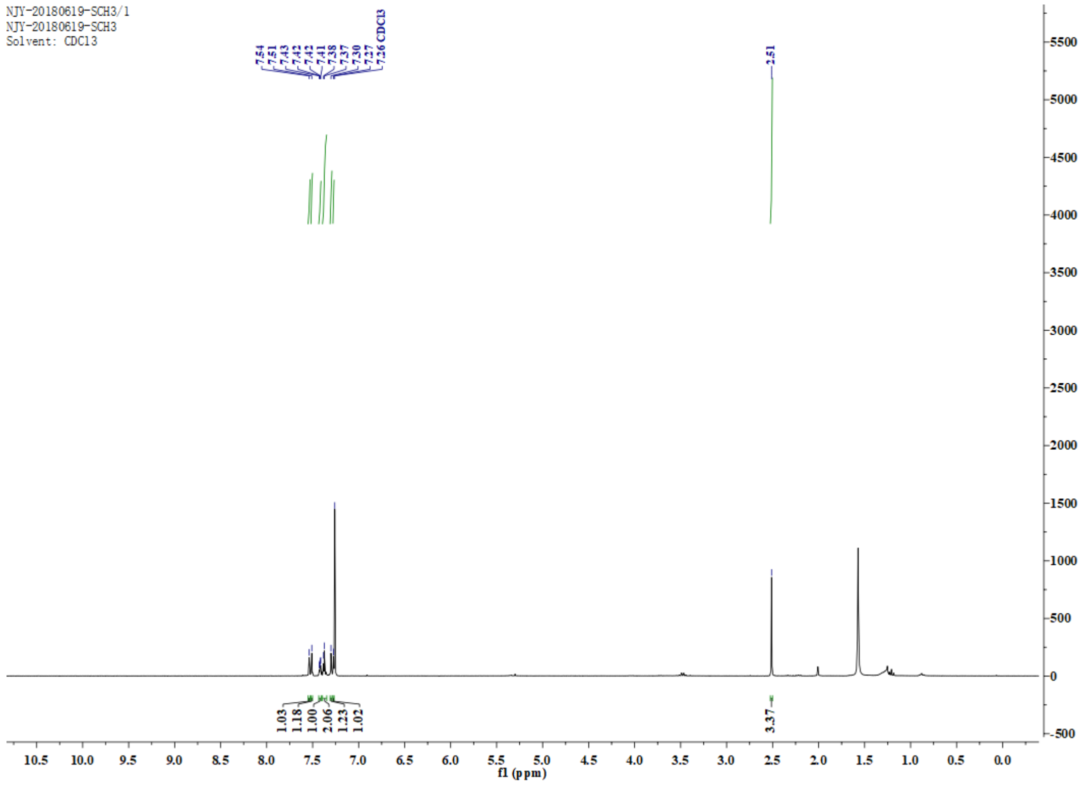

^{1}H NMR (300 MHz, $CDCl_3$) spectrum of 3-(4-(methylthio) phenyl) thiophene.

4-(thiophen-3-yl)benzenethiol (3) (TBT)

To a 250mL round bottom flask, the above 3-(4-(methylthio) phenyl) thiophene (**2**, 4 g, 19.2 mmol), Sodium 2-methyl-2-propanethiolate (*t*-BuSNa) (8.8 g, 76.8 mmol) and dry N,N-dimethylformamide (100 mL) were added, the reaction mixture was refluxed for 8 hours under nitrogen atmosphere. The solution was cooled and poured into hydrochloric acid solution (10 %， 100 mL), with the white precipitate subsequently formed. Then filtered and washed by flash chromatography, dried in vacuum oven to give the product **3** as pale yellow solid; yield 90 %. Please noticed the odor of *t*-BuSNa, all the processes should be carried on in the fume hood, and carbon powders were used to absorb residual odor. ^{1}H NMR (400 MHz, Chloroform-d) δ 7.47 (d, J = 8.5 Hz, 2H), 7.42 (dd, J = 2.9, 1.4 Hz, 1H), 7.38 (dd, J = 5.0, 2.9 Hz, 1H), 7.35 (dd, J = 5.0, 1.4 Hz, 1H), 7.30 (d, J = 8.5 Hz, 2H), 3.48 (s, 1H). ^{13}C NMR (101 MHz, Chloroform-d) δ 141.62, 133.59, 130.00, 129.45, 127.16, 126.46, 126.19, 120.21.

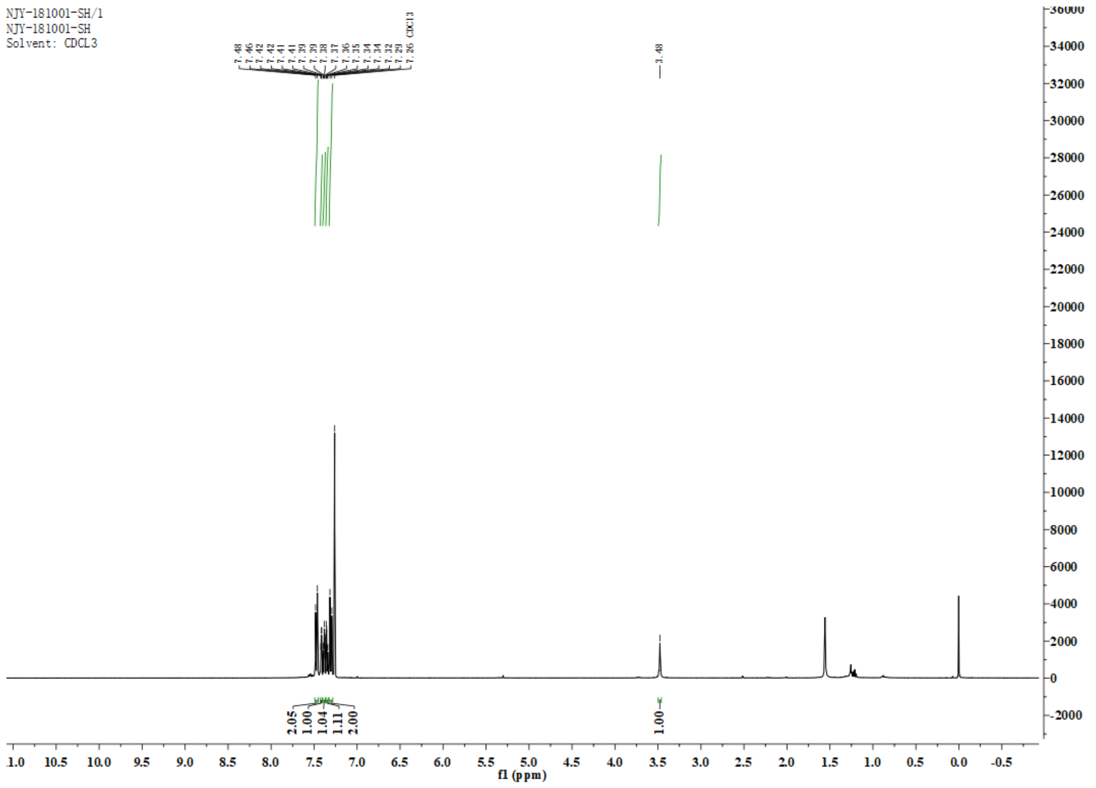

^{1}H NMR (400 MHz, $CDCl_3$) spectrum of 4-(thiophene-3-yl)benzenethiol.

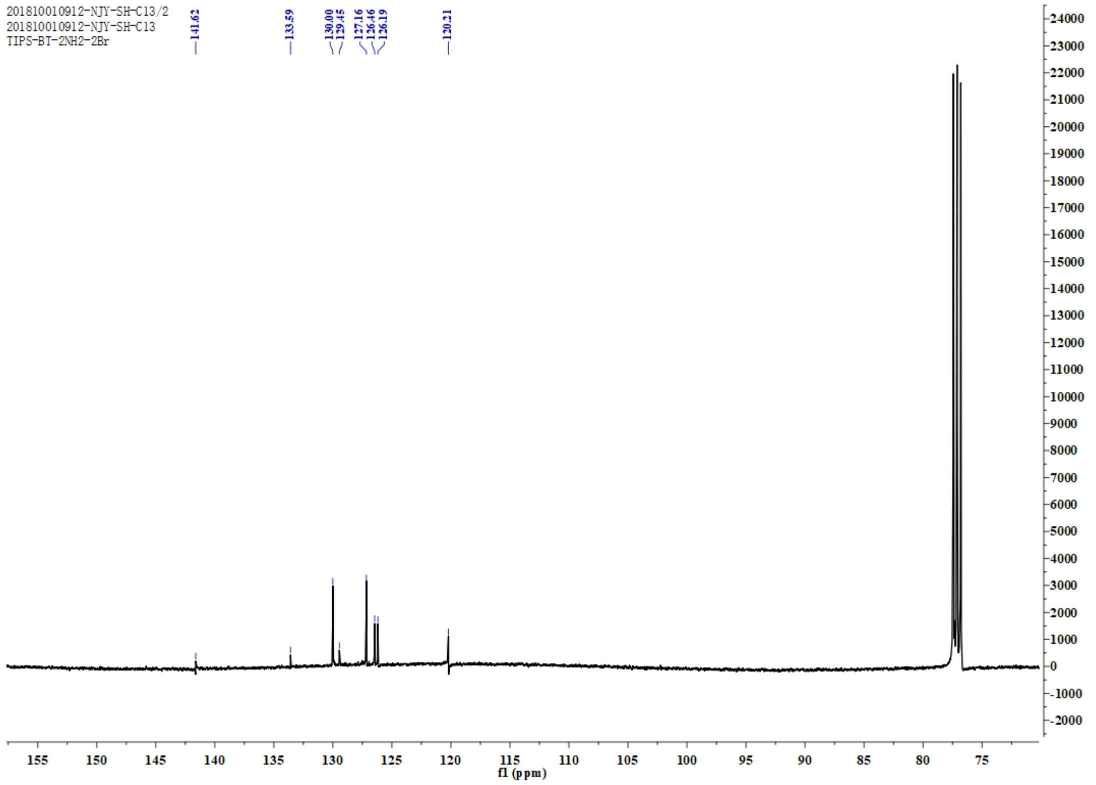

^{13}C NMR spectrum of 4-(thiophene-3-yl)benzenethiol.

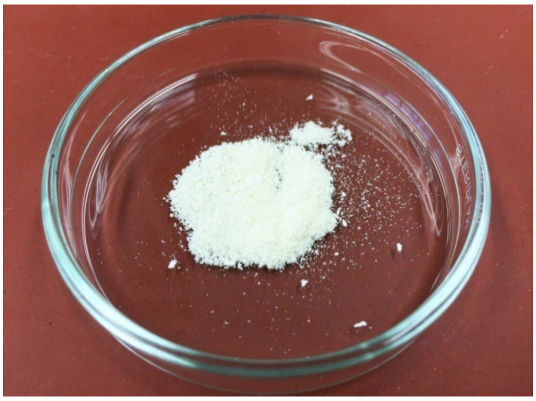

Figure S1. The photograph of the monomer TBT.

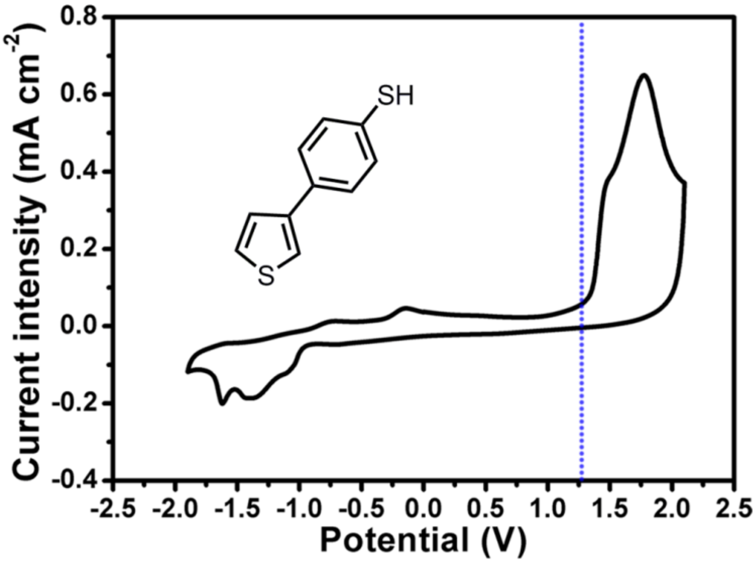

Figure S2. The CV curve of monomer TBT.

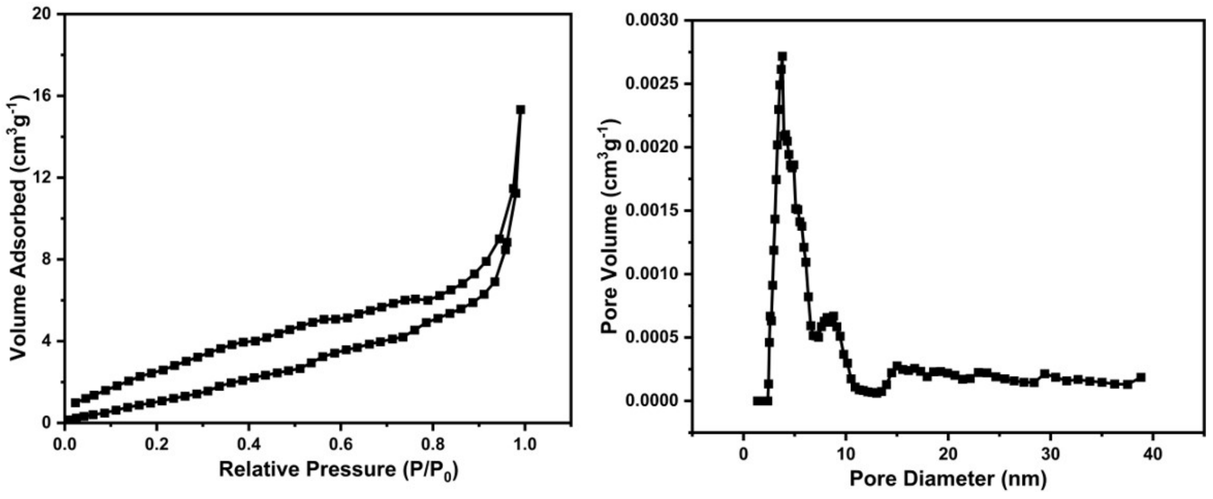

Figure S3. (a) BET Nitrogen adsorption and desorption test for the PTBT polymer, (b) the distribution of pore diameter using Barrett-Joyner-Halenda (BJH) method.

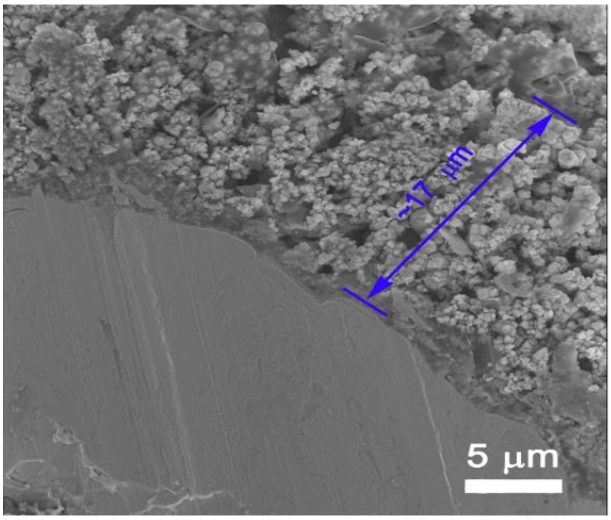

Figure S4. The cross-section SEM image of the S/PTBT@NF.

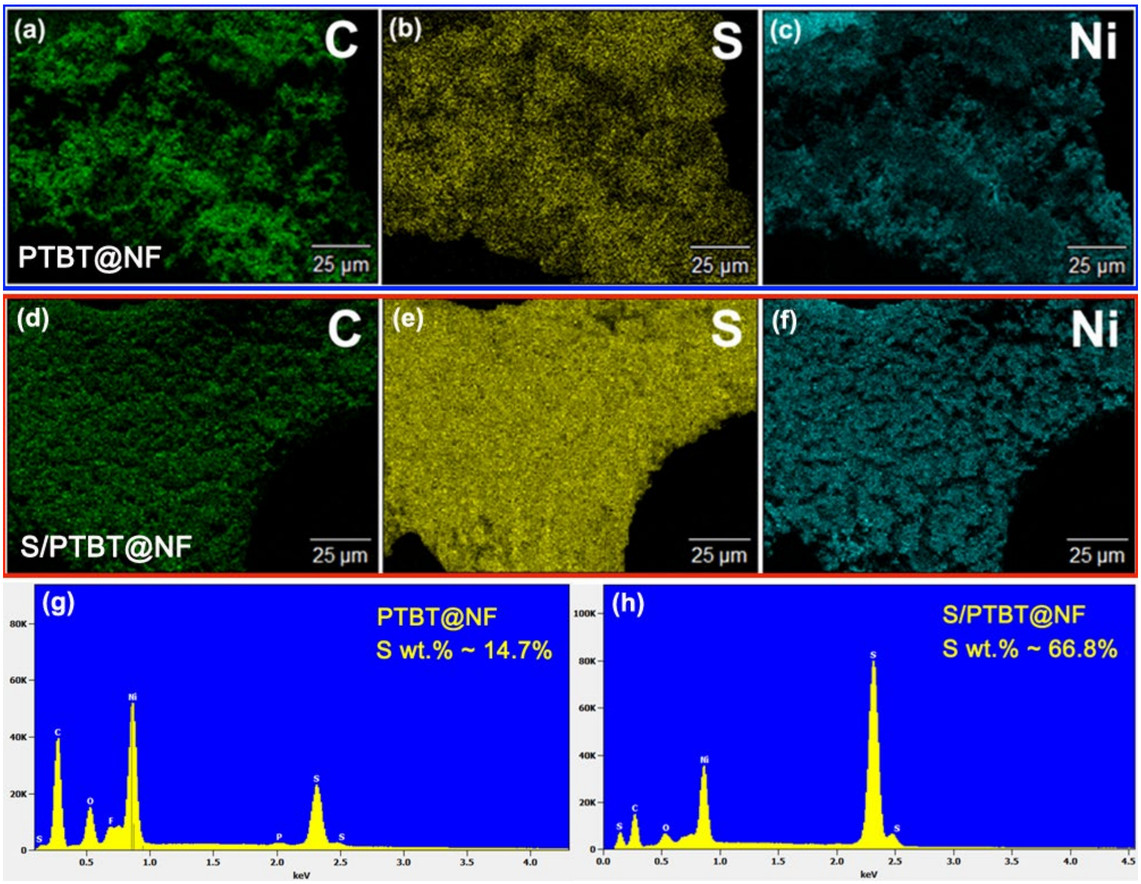

Figure S5. EDX of the PTBT@NF and S/PTBT@NF samples.

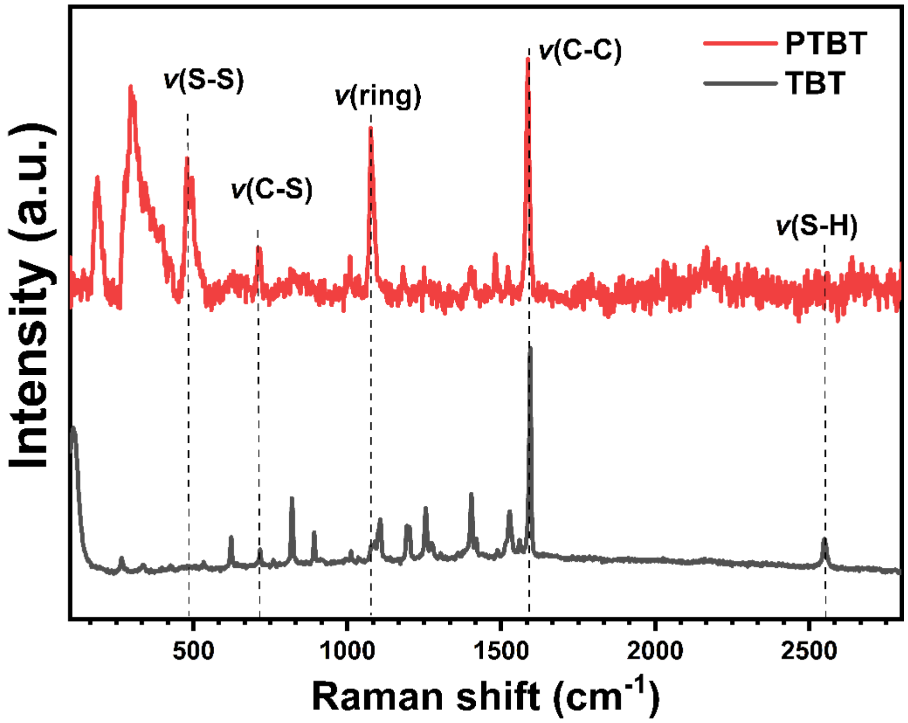

Figure S6. Raman spectra of monomer TBT and polymer PTBT.

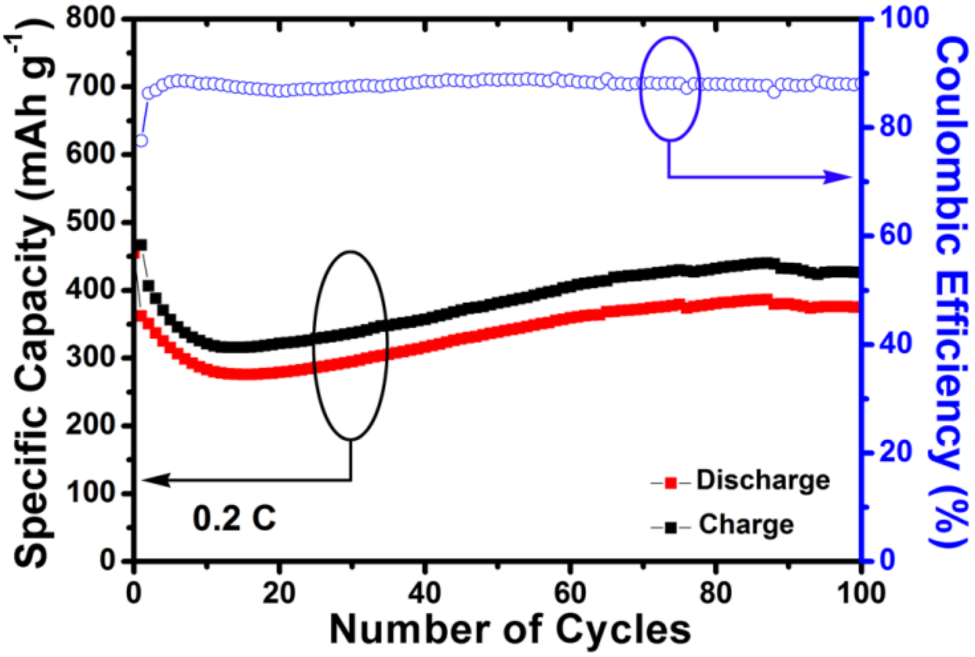

Figure S7 The discharge/charge capacities and columbic efficiencies of the control S&PTBT@NF cathode for 100 cycles at 0.2 C.

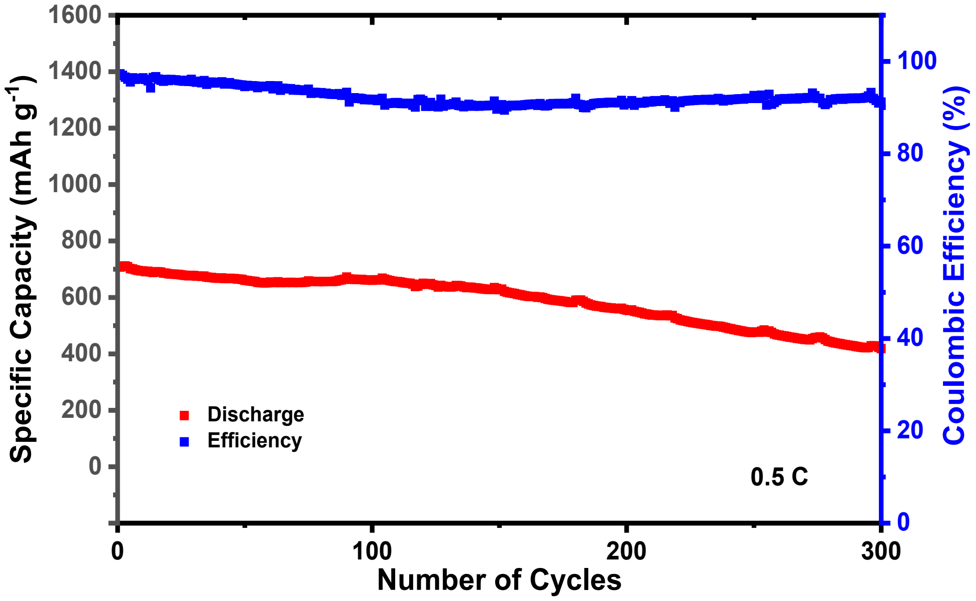

Figure S8 Long-term discharge profiles of the S/PTBT@NF cathode at 0.5 C rate.

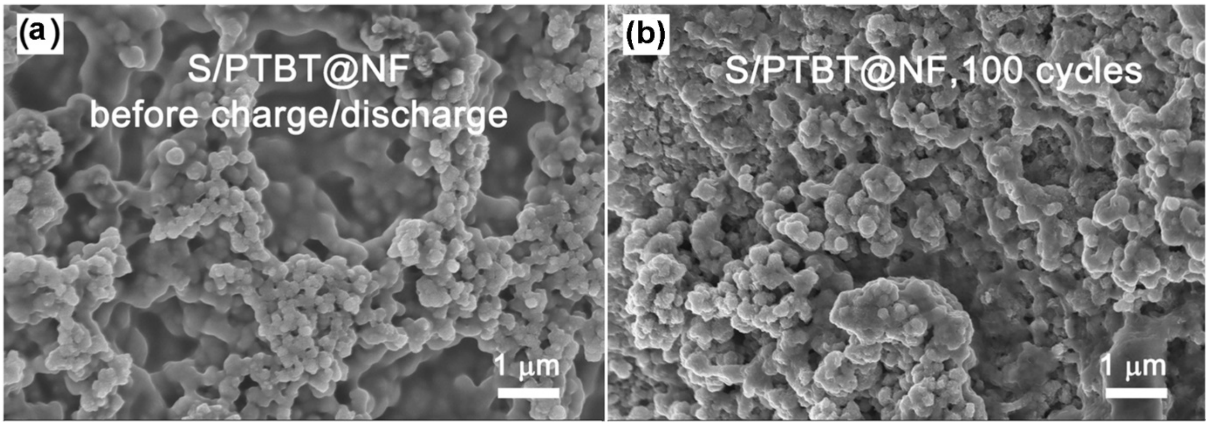

Figure S9 SEM of the S/PTBT@NF cathode before (a) and after (b) 100 charge/discharge cycles at 0.2 C.

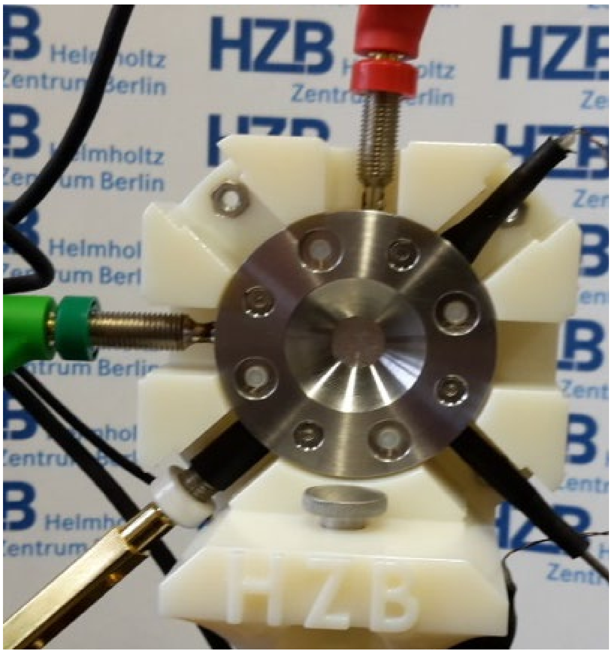

Figure S10. Image of the *operando* cell used for the X-ray imaging study.[7] The X-ray radiography images were recorded by using a laboratory CT setup.[8,9] The cell body consists of stainless steel and a polyether ether ketone (PEEK) ring. The inner geometry is comparable to that of a standard CR2032 coin cell.

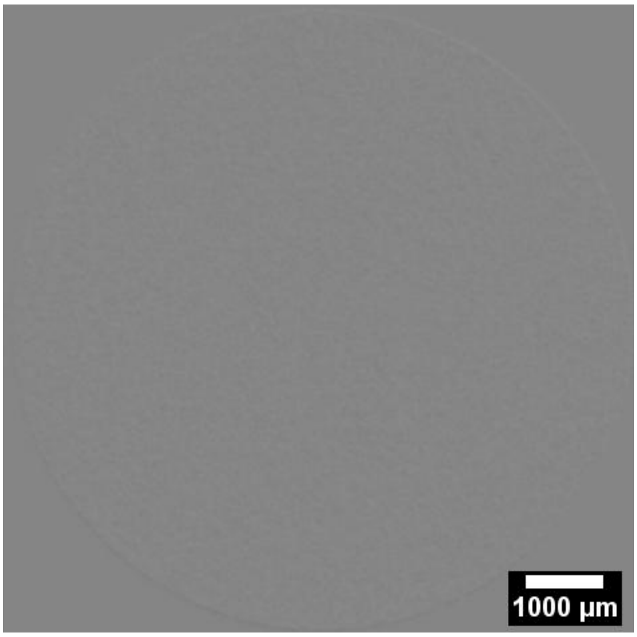

Video S1. The time-dependent morphological evolution of the particles during the period of the activity map.

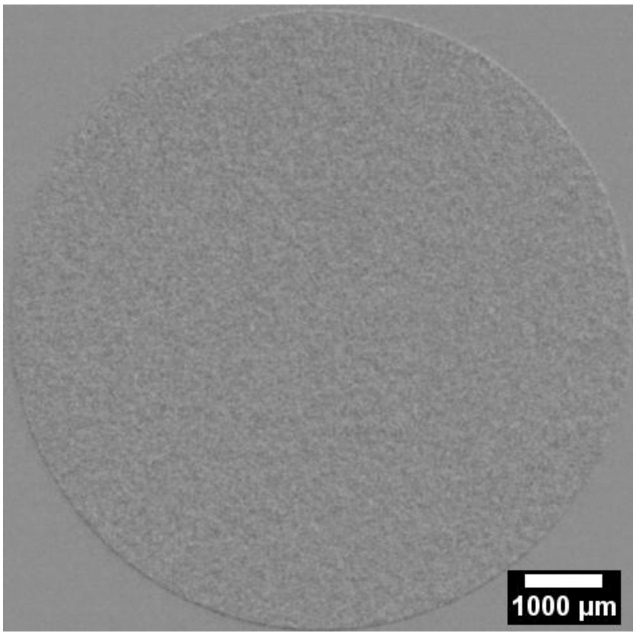

Video S2. The same time interval, which is extended to the final discharge of the third cycle.

S2. Theory calculations

For the generation of structures, a pre-relaxation was performed with the Avogadro software[1] employing the MMFF94 force field[2] using steepest descent minimization. Pre-relaxed structures were further optimized with DFT. All DFT calculations were performed using the all-electron, full-potential density functional theory package FHI-aims.[3] The exchange-correlation (XC) interactions were treated using the PBE0 functional together with the Tkatchenko-Scheffler method[1] to include van der Waals (vdW) interactions. FHI-aims-specific tier 2 basis sets and tight settings have been used. The convergence criterion for the total energy and for the forces was set to 10-6 eV and 10-5 eV/Å, respectively. The HOMO-LUMO gaps were deduced from the final relaxed structures. HOMO-LUMO gaps using the B3LYP[4,5] and PBE[6] XC functionals plus vdW interactions (B3LYP+vdW, PBE+vdW) were also computed for comparison (shown in **Figure S11**).

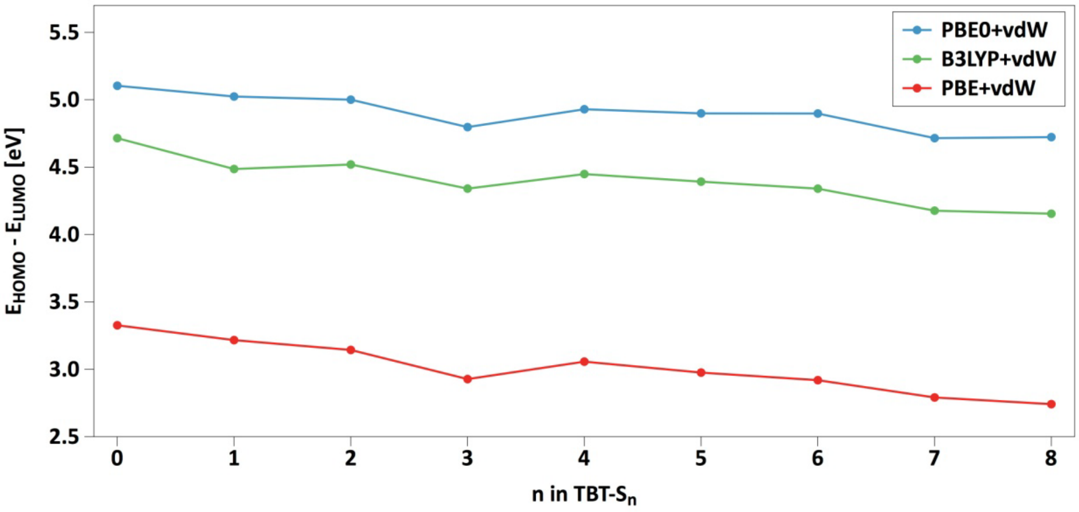

Figure S11 HOMO-LUMO gap of TBT and TBT-S_n monomers for *n* = 1, 2, …, 8 using different XC functionals. As expected, hybrid functionals show a larger HOMO-LUMO gap in comparison to PBE+vdW. All numbers correspond to the most stable configuration after optimization of the forces as described in the Methods section.

References:

[1] Hanwell, M. D.; Curtis, D. E.; Lonie, D. C.; Vandermeersch T.; Zurek, E.; Hutchison G. R. Avogadro: an advanced semantic chemical editor, visualization, and analysis platform**.** *Journal of Cheminformatics* **2012***, 4,* 17.

[2] Halgren T. A. Merck molecular force field. I. Basis, form, scope, parameterization, and performance of MMFF94. *Journal of Computational Chemistry* **1996***, 17,* 490-519.

[3] Blum, V.; Gehrke R.; Hanke, F.; Havu, P.; Havu, V; Ren, X; Reuter K; Scheffler, M. *Computer Physics Communications*, **2009**, *180*, 2175-2196.

[4] Becke, A. D. A new mixing of Hartree-Fock and local density-functional theories, *Journal of Chemical Physics*, **1993**, *98*, 5648-5652.

[5] Stephens P. J.; Devlin, F. J.; Chabalowski, C.F.; Frisch, M.J. *Ab Initio* Calculation of Vibrational Absorption and Circular Dichroism Spectra Using Density Functional Force Fields, *The Journal of Physical Chemistry*, **1994**, 98, 11623-11627.

[6] Perdew J. P.; Burke K.,; Ernzerhof, M. Generalized Gradient Approximation Made Simple, *Physical Review Letters,* **1997**, 78, 1396.

[7] Risse, S.; Hark, E.; Kent, B.; Ballauff, M. Operando Analysis of a Lithium/Sulfur Battery by Small-Angle Neutron Scattering. *ACS Nano* **2019**, *13*, 10233-10241.

[8] Kardjilov, N.; Hilger, A.; Manke, I.; Woracek, R.; Banhart, J. CONRAD-2: the new neutron imaging instrument at the Helmholtz-Zentrum Berlin. *Journal of Applied Crystallography* **2016**, *49*, 195-202.

[9] Kardjilov, N.; Hilger, A.; Manke, I. CONRAD-2: Cold Neutron Tomography and Radiography at BER II (V7). *Journal of Large-Scale Research Facilities* **2016**, *2* (DOI: 10.17815/jlsrf-2-108).
